# Targeting aerobic glycolysis combats tyrosine kinase inhibitor resistance of hepatocellular carcinoma

**DOI:** 10.1002/ijc.70091

**Published:** 2025-08-16

**Authors:** Longtao Zhao, Junjie Cheng, Yiming Zheng, Jing Wu, Jia Fan, Haixiang Sun, Chao Gao

**Affiliations:** ^1^ Department of Liver Surgery and Transplantation and Key Laboratory of Carcinogenesis and Cancer Invasion, Ministry of Education, Liver Cancer Institute Zhongshan Hospital, Fudan University Shanghai China; ^2^ Department of Radiation Oncology Zhongshan Hospital, Fudan University Shanghai China; ^3^ Graduate School of Bengbu Medical University Bengbu China; ^4^ Department of Medical Oncology Shanghai Geriatric Medical Center (Zhongshan Hospital, Fudan University Minhang Meilong) Shanghai China

**Keywords:** aerobic glycolysis, drug resistance, hepatocellular carcinoma, lactate, TKI

## Abstract

Hepatocellular carcinoma (HCC) represents the predominant form of primary liver cancer and is frequently identified at a late stage, necessitating systemic therapy. However, resistance to first‐line tyrosine kinase inhibitor therapies, such as sorafenib and lenvatinib, remains a significant clinical challenge. Recent research has revealed a strong link between aerobic glycolysis and drug resistance in HCC. Key enzymes in the glycolytic pathway, such as hexokinase, phosphofructokinase, and pyruvate kinase M, play central roles in the metabolic reprogramming of HCC cells. Aberrant activation of these enzymes not only promotes swift proliferation of tumor cells but also boosts adaptability. Lactate, the final product of glycolysis, is also pivotal in contributing to drug resistance in HCC. Moreover, signaling pathways, such as AMPK, HIF‐1, and c‐Myc, play key roles in tumor metabolic regulation, influencing energy balance, gene expression under hypoxia, and metabolic pathway control. These mechanisms interact synergistically, allowing HCC cells to endure and proliferate despite targeted therapies, ultimately resulting in drug resistance. Therefore, a deeper understanding of these metabolic and signaling regulatory mechanisms will help reveal the fundamental causes of drug resistance in HCC and provide new targets and directions for future therapeutic strategies.

## Graphical abstract

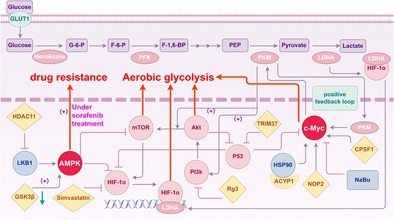

**Abbreviations**
AMP: adenosine monophosphate
AMPK: AMP‐activated protein kinase
ATP: adenosine triphosphate
BRCA1: breast cancer gene 1
CD147: cluster of differentiation 147
c‐Myc: cellular myelocytomatosis oncogene
CPSF6: cleavage and polyadenylation‐specific factor 6
CRC: colorectal cancer
DNA‐PKcs: DNA‐dependent protein kinase catalytic subunits
EGR1: early growth response 1
EMT: epithelial–mesenchymal transition
F‐2,6‐BP: fructose‐2,6‐bisphosphate
F‐6‐P: fructose‐6‐phosphate
FAO: fatty acid oxidation
GLUT1: glucose transporter protein type 1
GPR: G‐protein coupled receptor
HCC: hepatocellular carcinoma
HIF‐1α: hypoxia‐inducible factor 1‐alpha
HK: hexokinase
HSP90: heat shock protein 90
LDH: lactate dehydrogenase
LKB1: liver kinase B1
MAPK: mitogen‐activated protein kinase
MCTs: monocarboxylate transporters
mTOR: mammalian target of rapamycin
NBS1: Nijmegen breakage syndrome 1
PB2: proanthocyanidin B2
PD‐L1: programmed cell death ligand 1
PFK1: phosphofructokinase‐1
PFKFB3: 6‐phosphofructo‐2‐kinase/fructose‐2,6‐bisphosphatase 3
PHDs: prolyl hydroxylases
PI3K: phosphoinositide 3‐kinase
PK: pyruvate kinase
PKM2: pyruvate kinases type M2
PTMs: post‐translational modifications
STAT3: signal transducer and activator of transcription 3
TCA: tricarboxylic acid
TIGAR: TP53‐induced glycolysis regulatory phosphatase
TKIs: tyrosine kinase inhibitors
TME: tumor microenvironment
TRIM37: tripartite motif‐containing 37
VEGF: vascular endothelial growth factor

## 1 INTRODUCTION

The incidence of hepatocellular carcinoma (HCC), a leading cause of cancer‐related mortality globally, is expected to increase by 55% by 2040.^1^ With 70% of patients presenting at advanced stages due to nonspecific symptoms and rapid progression, systemic therapies remain critical when curative options are limited.^2^ First‐line tyrosine kinase inhibitors (TKIs) sorafenib and lenvatinib target angiogenesis and tumor proliferation through vascular endothelial growth factor (VEGF)/FGF receptor inhibition, but clinical efficacy is hampered by suboptimal responses and acquired resistance.^3^, ^4^ Only 30% benefit from sorafenib, with over 60% developing resistance within 1 year.^5^ Resistance mechanisms include genetic/epigenetic alterations, tumor microenvironment (TME) remodeling, and oncogenic pathway reactivation, but metabolic reprogramming has emerged as a key player. Tumor cells upregulate glycolysis to meet energetic and biosynthetic demands, supporting growth, survival, and drug resistance. Therefore, a deep understanding of metabolic reprogramming in HCC is crucial for developing new therapeutic strategies to overcome TKI resistance.

Glucose metabolism, a fundamental cellular process, is the subject of extensive research. Unlike normal cells, tumor cells metabolize glucose into lactate even under normoxic conditions, a phenomenon called aerobic glycolysis or the Warburg effect.^6^ This metabolic shift confers multiple advantages. Aerobic glycolysis elevates intracellular and extracellular lactate levels,^7^ which are exported via monocarboxylate transporters (MCTs), contributing to extracellular acidification and tumor progression. Simultaneously, glycolytic intermediates serve as precursors for biosynthetic pathways that produce amino acids, nucleotides, fatty acids, and glycogen.^8^ By supporting both the energetic and biosynthetic demands, aerobic glycolysis enables tumor cells to thrive in hostile TMEs and promotes rapid proliferation and survival.

Growing evidence indicates that aerobic glycolysis is pivotal in driving resistance targeted therapies, as both sorafenib and lenvatinib promote this metabolic shift.^9^, ^10^ This dynamic fosters tumor progression and complicates therapeutic strategies, as resistant cells continue to thrive and drive disease progression. In recent years, significant progress has been made in the identification of molecular targets of aerobic glycolysis with promising effects against HCC (Table 1). However, resistance mechanisms remain complex and incompletely understood. Thus, a thorough investigation into the metabolic basis of resistance may provide critical insights to advance research and improve treatment outcomes. This review aims to elucidate the mechanisms underlying glycolysis‐driven resistance of HCC to targeted therapies, particularly sorafenib and lenvatinib, by outlining the glycolytic process, TME, signaling pathways, and interactions, to highlight new perspectives for research on resistance to TKIs in clinical treatment.

**TABLE 1:** Promising markers and molecules in aerobic glycolysis and related targets in HCC.

Target	Marker or molecule	In vitro or in vivo	Involved factors
GLUT1	BAY‐876	Both	BAY‐876/GLUT1/HIF1α
	DHA	Both	DHA/YAP/GLUT1
	lncRNA SLC2A1‐AS1	Both	lncRNA SLC2A1‐AS1/STAT3/FOXM1/GLUT1
SGLT1	FLIPL	Both	FLIPL/SGLT1
HK2	HBx	Both	HBx/NF‐κBp65/HK2
	ASPP2	Both	ASPP2/WNT/β‐catenin/HK2
	miR‐125a	Both	miR‐125a/HK2
	miR‐885‐5p	Both	miR‐885‐5p/HK2
	miR‐202	Vitro	miR‐202/HK2
	CSN5	Vitro	CSN5/HK2
	UBR7	Both	UBR7/Keap1/Nrf2/Bach1/HK2
PFKL	EGR1	Both	EGR1/PFKL
	YTHDF3	Both	YTHDF3/PFKL
	miR‐338	Both	^125^I/miR‐338/PFKL
	A20		
PFKM	ZEB1	Both	ZEB1/PFKM
PKM2	PB2	Both	PB2/PKM2/HSP90/HIF‐1α
	TNFAIP6	Vitro	TNFAIP6/HNRNPC/c‐myc/PKM2
	SRSF7	Vitro	SRSF7/PKM2
	PWRN1	Both	PWRN1/PKM2/c‐Myc/LDHA
	USP35	Both	USP35/PKM2
	miR‐122	Vitro	miR‐122/PKM2
	TRIM35	Both	TRIM35/PKM2
	PARP14	Both	PARP14/JNK1/PKM2
	SHP‐1	Both	SHP‐1/SHP‐1
	PRMT6	Both	PRMT6/CRAF/MEK/ERK/PKM2
	Simvastatin	Both	Simvastatin/HIF‐1α/PPAR‐γ/PKM2
	MNX1‐AS1	Both	c‐Myc/MNX1‐AS1/PKM2
	GTPBP4	Both	GTPBP4/PKM2
LDHA	ACYP1	Both	ACYP1/HSP90/MYC/LDHA
	Circ_MAPK9	Both	Circ_MAPK9/miR‐642b‐3p/STAT3‐LDHA
	circUBE2D2	Both	circUBE2D2/miR‐889‐3p/LDHA
	miR‐142‐3p	Vitro	miR‐142‐3p/LDHA
	CircFOXK2	Both	circFOXK2/FOXK2‐142aa/LDHA
HIF1‐α	ACE2	Vitro	ACE2/Ang‐(1–7)/Mas receptor/ROS/HIF1α
	miR‐592	Both	miR‐592/WSB1/HIF‐1α
	MiR‐199a‐5p	Vitro	MiR‐199a‐5p/HIF‐1α
	Genistein	Both	Genistein/HIF‐1α/GLUT1 and HK2
	HMGB1	Both	HMGB1/YAP/HIF1α
	miR‐3662	Both	miR‐3662/HIF‐1α
	COX2	Vitro	COX2/HIF1α/PKM2
	Metformin	Vitro	Metformin/HIF‐1α/PFKFB3/PFK1
	HBXIP	Vitro	HBXIP/METTL3/HIF‐1α
	USP29	Both	USP29/HIF1α
	TFB2M	Both	TFB2M/NAD^+^/SIRT3/HIF‐1α
	O‐GlcNAcylation	Both	O‐GlcNAcylation/HIF‐1α
c‐Myc	NOP2	Both	MAZ/NOP2/c‐Myc
	ACTR	Both	ACTR/c‐Myc
	CPSF6	Both	CPSF6/c‐Myc
	METTL5	Both	p300/CREB1/METTL5/USP5/c‐Myc
	NaBu	Both	NaBu/c‐myc/HK2
AMPK	TUG1	Both	TUG1/miR‐455‐3p/AMPKβ2/HK2
	Cordycepin	Vitro	Cordycepin/AMPK/Akt
	SCT‐1015	Both	AMPK/HIF‐1α
PI3K/Akt	CD36	Both	CD36/Src/PI3K/Akt/mTOR
	RBBP7	Both	SP1/RBBP7/PI3K/Akt
	RPLP2	Both	RPLP2/TLR4/PI3K/Akt/HIF1α
	VersicanV1	Both	VersicanV1/EGFR/PI3K/AKT
	SSL6	Both	SSL6/CD47/PI3K/Akt/HIF1α
	PTEN	Both	PTEN/PI3K/Akt
	miR‐30a‐5p	Both	miR‐30a‐5p/CLCF1/PI3K/Akt
Akt	Compound K	Vitro	Compound K/AKT/mTOR/c‐Myc
	CircRPN2	Both	CircRPN2/ENO1/Akt/mTOR
mTOR	B3galt5	Both	b3galt5/mTOR/p70s6k
P53	CD147	Vitro	PI3K/Akt/MDM2
	TRIM37	Both	TRIM37/P53

## 2 TARGETING KEY ENZYMES IN GLYCOLYTIC METABOLISM IN HCC

**FIGURE 1:**
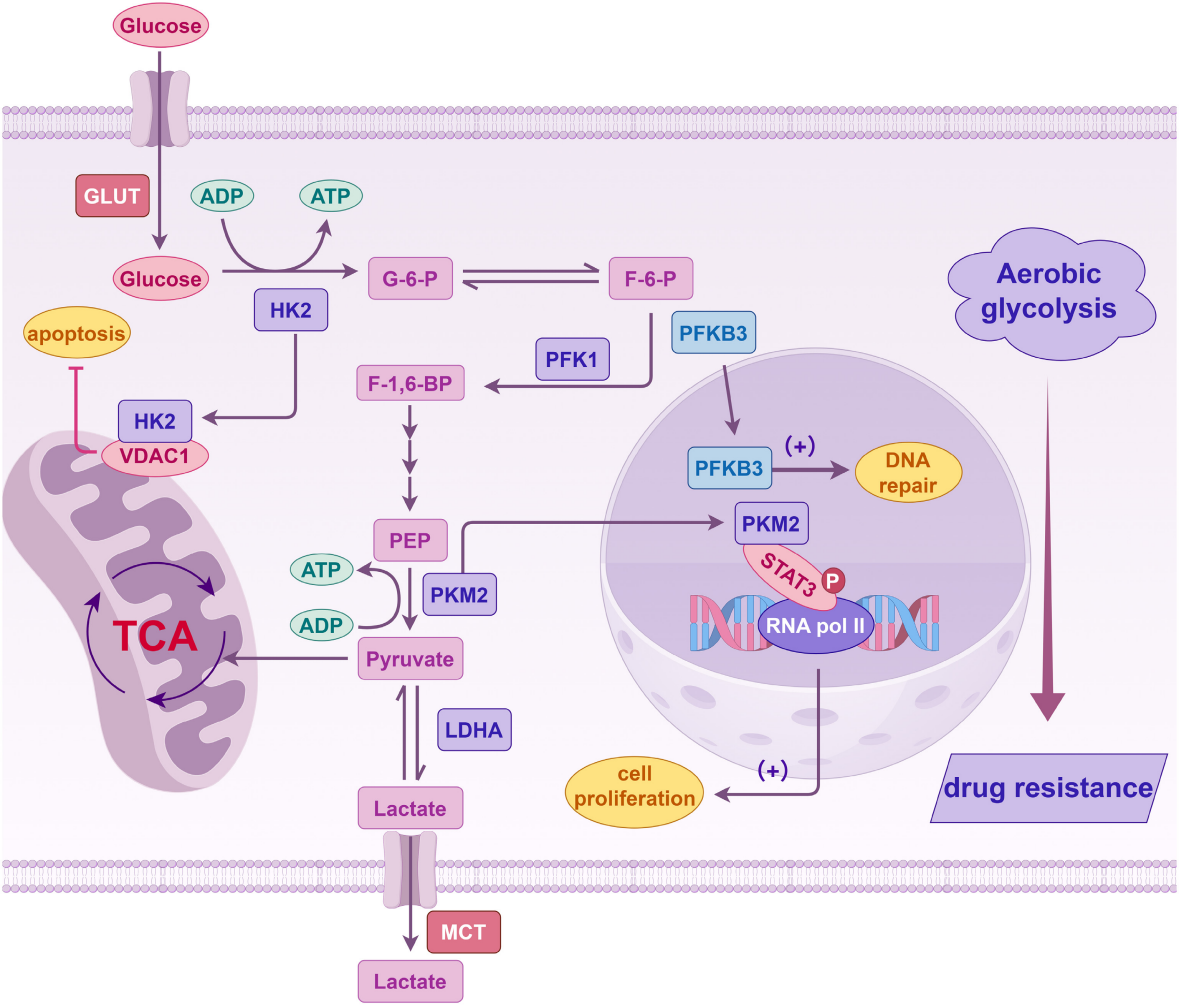
Key steps and essential enzymes involved in aerobic glycolysis. HK2 enhances glycolytic flux and promotes cell survival by inhibiting apoptosis. PFK1 regulates the conversion of F‐6‐P to fructose‐1,6‐bisphosphate, while PFKFB3 serves as a powerful allosteric activator of PFK1 and functions in both cytoplasmic and nuclear roles. PKM2 exists in both active and inactive forms, supporting the Warburg effect, while also influencing transcriptional regulation and apoptosis evasion. Collectively, these enzymes are vital to understand and address drug resistance in HCC.

Glycolysis is a multi‐step process where glucose is initially broken down into pyruvate, regulated by the key enzymes hexokinase 2 (HK2), phosphofructokinase‐1 (PFK1), and pyruvate kinase type M2 (PKM2).^11^ The specific roles of these enzymes in metabolic reprogramming of HCC and involvement in inducing resistance to targeted therapies are detailed below (Figure 1).

### 2.1 Hexokinase 2

Hexokinase (HK) initiates glycolysis by phosphorylating glucose to glucose‐6‐phosphate. Among the five identified human hexokinase isoenzymes,^12^ HK2 is abundantly expressed in HCC and directly correlated with pathological staging and poor prognosis.^13^ Beyond its role in glucose metabolism, HK2 promotes tumor survival by inhibiting apoptosis. On the outer mitochondrial membrane, HK2 binds to voltage‐dependent anion channel protein 1, facilitating adenosine triphosphate (ATP) from mitochondria to the cytosol^14^ and interfering with pro‐apoptotic Bcl‐2‐associated protein x, thereby supporting cancer cells survival. Given its central role in metabolism and survival, HK2 has been implicated in HCC. Silencing HK2 can enhance sorafenib sensitivity and suppress tumor growth in vivo.^15^ Additionally, the glucose analog 2‐deoxy‐D‐glucose non‐competitively inhibits HK2, suppressing HCC cell proliferation and synergizes with sorafenib to enhance anti‐tumor efficacy.^16^ Systemically administered drugs typically accumulate in the liver, and since HK2 is predominantly expressed in HCC, even modest doses of HK2 inhibitors can specifically target tumor cells with minimal impact on normal liver cells.^17^ Therefore, precise HK2‐targeted strategies offer promising potential to overcome drug resistance in HCC.

### 2.2 PFK1 and PFKFB3

PFK1 is the second key regulatory enzyme involved in glycolysis, using ATP to catalyze the conversion of fructose‐6‐phosphate (F‐6‐P) to fructose‐1,6‐bisphosphate. PFKL is one of the three PFK1 isoforms and is predominantly expressed in the liver, where it plays a central role in glycolytic reprogramming and HCC progression.^18^ Early growth response 1 (EGR1), a zinc finger transcription factor, binds to the PFKL promoter, repressing transcription and inhibiting glycolysis in HCC.^19^ Reduced EGR1 levels correlate with sorafenib resistance, whereas EGR1 restoration enhances sensitivity both in vitro and in xenografts. Similarly, the E3 ubiquitin ligase A20 promotes PFKL degradation, impairing glycolysis and suppressing HCC proliferation.^20^

In parallel, 6‐phosphofructo‐2‐kinase/fructose‐2,6‐bisphosphatase 3 (PFKFB3) produces fructose‐2,6‐bisphosphate (F‐2,6‐BP), the most powerful allosteric activator of PFK‐1, driving high glycolytic flux in tumors. PFKFB3 is overexpressed in multiple cancers, including breast, colon,^21^ and HCC, where it correlates with larger tumor burden and poorer prognosis.^22^ PFKFB3 functions both in the cytoplasm, regulating glycolysis, and in the nucleus,^23^ where it promotes cell proliferation via cyclin‐dependent kinase 1‐mediated p27 phosphorylation and suppresses apoptosis. In HCC, PFKFB3 activates the protein kinase B (Akt)/excision repair cross complementation group 1 pathway, enhancing DNA repair and tumor growth.^22^ Moreover, sorafenib treatment induces PFKFB3 expression, contributing to drug resistance through a PFKFB3/hypoxia‐inducible factor 1‐alpha (HIF‐1α) feedback loop.^24^ Interestingly, PFKFB3 inhibition can redirect glucose to the pentose phosphate pathway, potentially generating alternative resistance mechanisms.^25^ Future research is needed to determine whether dual blockade of both pathways may improve therapeutic efficacy.

### 2.3 Pyruvate kinase M2

As the final key enzyme, pyruvate kinase (PK) catalyzes the conversion of phosphoenolpyruvate and adenosine diphosphate to pyruvate and ATP. Among its four isoforms,^26^ PKM2 is commonly overexpressed in cancers and promotes tumor proliferation and metastasis. PKM2 exists in two forms: a glycolytically active tetramer and an inactive dimer. The tetramer supports the Warburg effect by facilitating glucose flux toward lactate production, sustaining tumor metabolism.^27^ In HCC, PKM2 promotes chemoresistance through increased glycolytic flux.

Beyond its metabolic role, dimeric PKM2 translocates to the nucleus, where it functions as a protein kinase, regulating gene transcription by phosphorylating transcription factors and histones. For example, PKM2 phosphorylates signal transducer and activator of transcription 3 (STAT3) at Tyr105, activating transcription of mitogen‐activated protein kinase kinase 5 and promoting cell proliferation. Aberrant STAT3 activation is also implicated in drug resistance, such as gefitinib resistance in colorectal cancer (CRC).^28^ In HCC, silencing PKM2 restores sorafenib sensitivity in resistant Hep3BSR and LM3‐SR cells.^29^ Similarly, the flavonoid proanthocyanidin B2 (PB2) reduces lactate production by downregulating PKM2, enhancing sorafenib efficacy in xenograft models.^27^ Although the nuclear functions of PKM2 remain incompletely understood, targeting its nuclear translocation with small‐molecule inhibitors represents a promising therapeutic approach for overcoming resistance.

### 2.4 Metabolic crosstalk of aerobic glycolysis in HCC

In TKI‐resistant HCC, aerobic glycolysis is not an isolated process but functionally interlinked with glutamine metabolism^30^ and fatty acid oxidation (FAO).^31^ Under glucose‐limiting conditions or glycolytic inhibition, glutamine catabolism is upregulated to maintain TCA cycle flux and biosynthetic output. Simultaneously, enhanced FAO supplies acetyl‐CoA and NADPH to support ATP generation, redox balance, and histone acetylation—crucial for maintaining proliferation and resisting therapeutic stress. These compensatory pathways ensure metabolic continuity when glycolysis is compromised.

Such interactions are not merely additive but deeply integrated. For example, glycolytic activation can increase glutamine uptake (e.g., via SLC1A5), while α‐ketoglutarate derived from glutamine feeds back to influence glycolytic enzyme transcription via epigenetic regulation. Similarly, FAO supports the high ATP demand of glycolysis and contributes to lipogenesis, which in turn replenishes substrates for FAO. This reciprocal reinforcement suggests that TKI‐resistant HCC cells operate through a flexible metabolic network rather than single‐pathway dominance. Therefore, breaking this metabolic plasticity may require dual‐ or multi‐pathway targeting. A nuanced understanding of when and how these pathways compensate for each other is key to identifying vulnerabilities in resistant tumors.

## 3 TARGETING LACTATE METABOLISM

**FIGURE 2:**
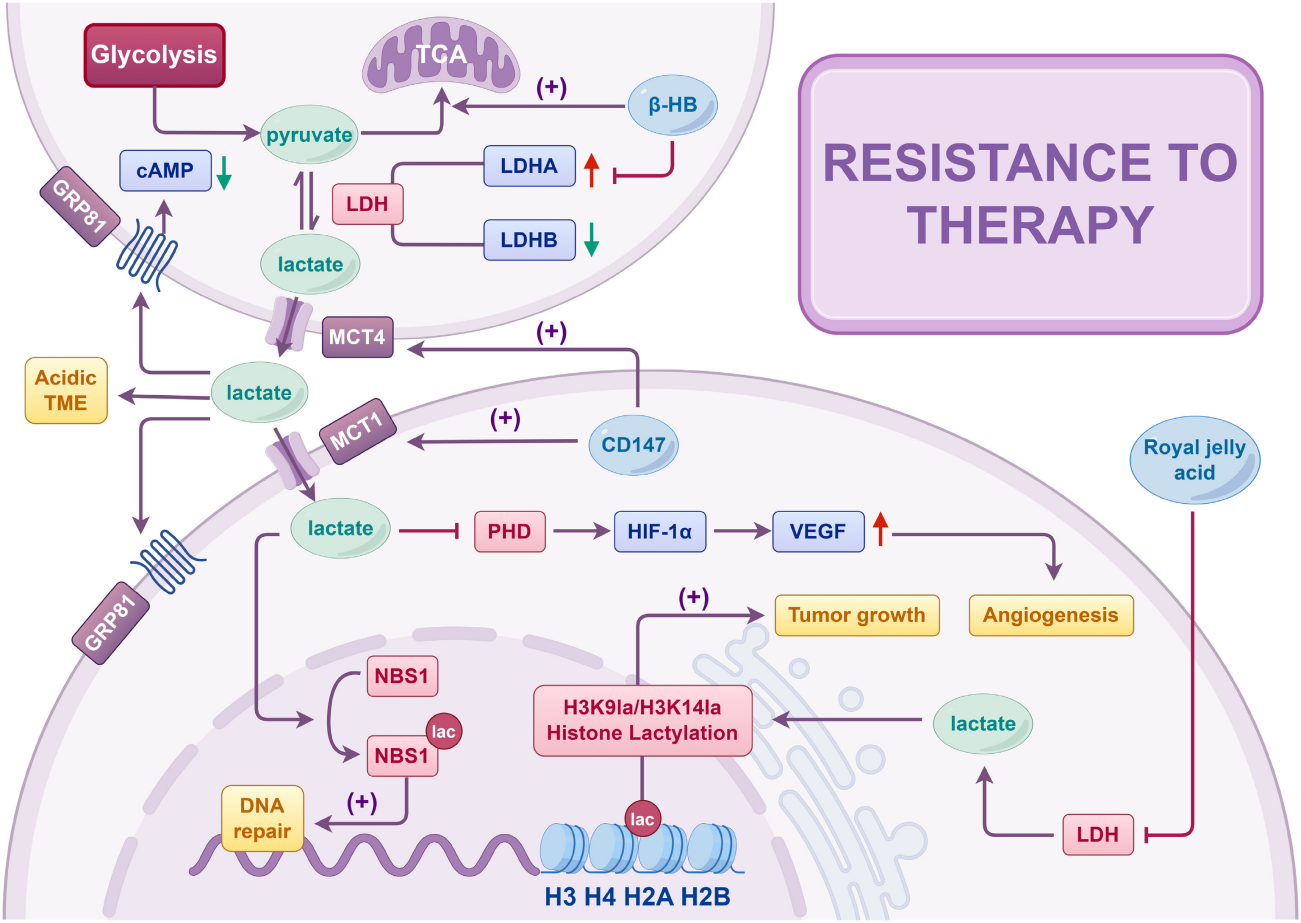
Lactate is an important metabolic regulator in HCC. Key enzymes, like LDHA, enhance glycolysis, while low LDHB levels sustain high lactate, correlating with poor prognosis. MCTs facilitate lactate transport, contributing to an acidic TME and chemotherapy resistance. Additionally, lactate activates GPR81, affecting immune responses and DNA repair. Lactylation further modifies proteins, impacting metabolic pathways. Understanding these mechanisms may lead to the development of novel targeted therapies for HCC.

Previously regarded as a metabolic byproduct of hypoxia, lactate is now recognized as a key metabolic regulator involved in tumor progression, metastasis, prognosis, and survival in HCC.^32^ To elucidate its role in drug resistance, four key aspects were examined: lactate production and transport, lactate‐mediated signaling mechanisms, and lactylation modifications. A comprehensive understanding of these mechanisms may reveal how lactate facilitates resistance through diverse pathways (Figure 2).

### 3.1 Lactate dehydrogenase

The transformation of pyruvate to lactate is facilitated by lactate dehydrogenase (LDH), a heterotetrameric enzyme consisting of A and B subunits.^33^ LDH‐A enhances pyruvate‐to‐lactate conversion, while LDH‐B favors pyruvate oxidation to acetyl‐CoA for entry into the Krebs cycle. Dysregulated LDH expression, particularly upregulation of LDHA and downregulation of LDHB, promotes tumor progression. LDHA activity is essential for aggressive tumor growth^34^ and has emerged as a promising therapeutic target. In HCC, the circular RNA circUBE2D2 promotes glycolysis and sorafenib resistance via the miR‐889‐3p/LDHA axis.^35^ Similarly, exogenous β‐hydroxybutyrate, a ketone body produced during ketogenesis, reduces lactate production and restores sorafenib sensitivity by downregulating LDHA expression.^36^

While LDHA's role in tumor metabolism is well‐documented, LDHB functions appear more context‐complex. LDHB primarily converts lactate back to pyruvate, promoting oxidative phosphorylation. In HCC, suppressed LDHB expression sustains high lactate levels and aerobic glycolysis, contributing to rapid tumor growth.^37^ Bioinformatic analysis of The Cancer Genome Atlas cohort demonstrates that a low LDHB/LDHA ratio correlates with poor prognosis in HCC. However, in lung and breast cancers,^38^ elevated LDHB expression is associated with enhanced invasiveness and metastasis, suggesting a dual, tissue‐specific role. Therefore, further research into the regulatory mechanisms and functional roles of LDHB in HCC may inform novel metabolic‐targeted therapies.

### 3.2 Monocarboxylate transporters

Due to hydrophilicity and weak acidity, MCT family proteins are required for transmembrane transport of lactate. Encoded by the solute carrier family 16, MCTs comprise 14 members with similar basic structures.^39^ Among these, only the membrane‐bound proton‐coupling isoforms MCT1, MCT2, MCT3, and MCT4 facilitate lactate transport across the cell membrane. As the most studied in cancer, MCT1 and MCT4 are frequently overexpressed in many types of cancer, ranging from solid tumors to hematological malignancies, including HCC.^39^, ^40^ MCT4 mediates lactate efflux, preventing intracellular acidification and maintaining acidity in the TME, which fosters tumor growth, invasion, and metastasis. Conversely, MCT1 mediates lactate uptake into adjacent cells, activating oncogenic signaling pathways that promote tumor progression, immune evasion, and chemoresistance.^41^ Thus, targeting MCTs to disrupt lactate homeostasis may impair tumor metabolic adaptation and offer novel therapeutic opportunities for HCC. Notably, curcumin has been reported to reverse lactate‐induced chemoresistance by modulating MCT1 expression, underscoring the therapeutic relevance of MCT targeting in HCC management.^42^

In addition to MCTs themselves, their trafficking to the plasma membrane requires the chaperone protein cluster of differentiation 147 (CD147), which facilitates the proper localization and functional activity of both MCT1 and MCT4.^43^ Functional inhibition of CD147 may interfere with the localization and function of MCTs, thereby further inhibiting lactate transport and enhancing anti‐cancer efficacy. These findings provide new perspectives for HCC treatment and guide future research directions.

### 3.3 Lactate‐mediated signaling transduction

Beyond its role as a metabolic byproduct, lactate functions as a critical signaling molecule that promotes tumor progression through both intracellular and extracellular mechanisms. Intracellularly, lactate inhibits prolyl hydroxylase 2, stabilizing HIF‐1α,^44^ and promoting VEGF expression. Additionally, tumor‐derived lactate activates the MCT1/nuclear factor‐κB/cyclooxygenase‐2 pathway, inducing neutrophil expression of programmed cell death ligand 1 (PD‐L1), which reduces T cell cytotoxicity and decreases lenvatinib efficacy.^45^ This mechanism indicates lactate's role in fostering an immunosuppressive TME and facilitating immune evasion. Given lactate's ability to induce PD‐L1 expression, combining lenvatinib with PD‐1/PD‐L1 checkpoint inhibitors might enhance antitumor immune responses and improve therapeutic outcomes.

The extracellular signaling function of lactate involves activation of the G‐protein coupled receptor (GPR) 81. Besides HCC, GPR81 is highly abundant in cancers of the colon, rectum, breast, lung, cervix, and pancreas.^46^, ^47^ Upon engagement by lactate, GPR81 upregulation promotes tumorigenic phenotypes via both autocrine and paracrine mechanisms. In lung cancer, GPR81 enhances PD‐L1 expression via the Wwtr1‐encoded transcriptional coactivator pathway, contributing to immune escape.^48^ Paracrine activation of GPR81 on dendritic cells impairs antigen presentation by downregulating major histocompatibility complex class II expression.^49^ Additionally, GPR81 also induces DNA repair proteins, such as the DNA‐dependent protein kinase catalytic subunits nibrin and breast cancer gene 1, facilitating drug resistance.^50^, ^51^

Given the multifaceted role of GPR81 in immune escape, DNA repair, and therapy resistance, it represents a promising therapeutic target in HCC. Development of specific GPR81 inhibitors may effectively suppress tumor progression and sensitize HCC to therapies. Alternatively, modulation of extracellular lactate levels or inhibition of lactate transporters such as MCT1 to prevent lactate–GPR81 interaction may also provide viable therapeutic avenues. Nevertheless, further mechanistic studies are warranted to fully elucidate the role of GPR81 in HCC and optimize its therapeutic targeting.

### 3.4 Lactylation

Post‐translational modifications (PTMs) are essential for diverse biological processes, including gene expression, signaling, metabolism, proliferation, migration, and tumor invasion. Among these, protein lactylation, as first reported by Zhao et al.,^52^ represents a significant addition to the repertoire of PTMs following phosphorylation, acetylation, methylation, and ubiquitination. Lactylation involves the covalent attachment of a lactyl group to lysine residues, modulating protein function, stability, and interactions. This modulation is dynamically orchestrated by specific enzymes termed “writers” and “erasers.” For instance, the early region 1A‐associated protein p300 is a lactylation writer, and overexpression increases histone lactylation levels.^53^ Conversely, histone deacetylases can remove lactylation. Notably, sirtuin 3, a nicotinamide adenine dinucleotide‐dependent deacetylase, can remove lactylation from non‐histone proteins, thereby suppressing HCC progression.^54^

Lactylation levels are closely linked to glycolytic activity and intracellular lactate accumulation, which can modulate the function of key metabolic enzymes. For example, LDHA‐deficient macrophages show reduced lactate production and histone lactylation.^52^ In non‐small cell lung cancer, lactylation downregulates HK1 and PKM expression to preserve mitochondrial function.^55^ In HCC, enrichment analyses have demonstrated that lactylation significantly impacts metabolic enzymes such as dehydrogenases and oxidoreductases, ultimately affecting cellular metabolism and energy homeostasis.^56^

Beyond metabolism, lactylation plays important roles in tumor biology. Histone lactylation is significantly elevated in HCC tissues compared to adjacent non‐tumorous tissues,^57^ particularly H3K9 and H3K56, and its inhibition—via lactate reduction—suppresses tumorigenicity. Royal jelly acid similarly reduces lactate levels and histone lactylation (H3K9 and H3K14), leading to decreased proliferation and migration while promoting apoptosis.^58^ Intriguingly, lactylation also promotes rapid DNA repair. For instance, lactylation of Nijmegen breakage syndrome 1 (NBS1) enhances DNA repair capacity, contributing to drug resistance.^59^ These findings highlight lactylation as a key mechanism in HCC therapeutic resistance. However, further mechanistic studies are warranted to elucidate the precise pathways by which lactylation modulates resistance.

## 4 TARGETING UPSTREAM MECHANISTIC PATHWAYS

**FIGURE 3:**
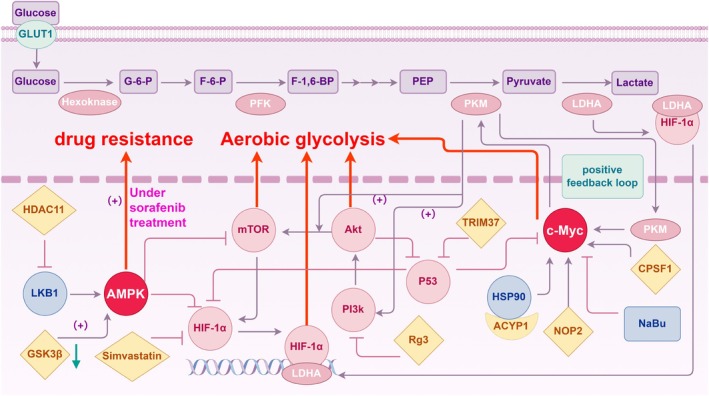
The complex regulatory mechanisms and factors involved in aerobic glycolysis in HCC. AMPK typically inhibits glycolysis but it can promote resistance under therapeutic stress. c‐Myc enhances glycolysis, contributing to energy supply and resistance to treatments, like sorafenib. HIF‐1 drives glycolysis under hypoxic conditions, leading to worsening prognosis and treatment resistance. The PI3K/Akt pathway boosts tumor growth and survival through glycolysis and it is associated with multidrug resistance. As a crucial tumor suppressor, p53 inhibits glycolysis, but is often dysfunctional in tumors, further promoting resistance.

Tumorigenesis involves genetic alterations, including proto‐oncogene mutations, tumor suppressor gene inactivation, and abnormal transcription factors expression. In HCC, these molecular changes not only promote tumor formation and progression, but are closely associated with metabolic reprogramming.^60^ A deeper understanding of these molecular regulators can clarify how tumor metabolism contributes to therapeutic resistance. The focus will now shift to the specific mechanisms of mitogen‐activated protein kinase (MAPK), the cellular myelocytomatosis oncogene (c‐Myc), HIF‐1, the phosphoinositide 3‐kinase (PI3K)/Akt/mammalian target of rapamycin (mTOR) signaling pathway, and p53 in targeted drug resistance in HCC (Figure 3).

### 4.1 AMP‐activated protein kinase

AMP‐activated protein kinase (AMPK) is a highly conserved heterotrimeric serine/threonine (Ser/Thr) protein kinase complex that plays a central role in regulating cellular energy homeostasis.^61^ Its activation is triggered by increased intracellular AMP/ATP or ADP/ATP ratios, leading to phosphorylation at Thr172 by upstream kinases. Liver kinase B1 (LKB1), a tumor suppressor, is a primary upstream activator linking AMPK to cancer metabolism.^62^ Alternatively, calcium/calmodulin‐dependent protein kinase kinase 2 can activate AMPK independently of energy status.^63^ Notably, phosphorylated AMPK levels are reduced in HCC tissues compared to adjacent non‐tumor tissues,^64^ and low AMPK activity correlates with poor survival outcomes.

The role of AMPK in glycolysis is complex and content‐dependent. On one hand, AMPK activation suppresses aerobic glycolysis and tumor growth by inhibiting the mTOR/GSK3β axis, leading to downregulation of key glycolytic regulators including GLUT1, HK2, PFKFB3, and PKM2.^65^ AMPK also influences HIF‐1α activity, with compounds like SCT‐1015 reducing glycolysis and enhancing oxidative phosphorylation via the AMPK/HIF‐1α pathway.^66^ Upstream inhibition of AMPK through oncogenic regulators such as histone deacetylase 11, which suppresses LKB1, promotes glycolysis and contributes to cancer stem cell maintenance and sorafenib resistance.^67^ Interestingly, under nutrient deprivation, AMPK activation can preserve cell viability, whereas inhibition of AMPK under these conditions may sensitize tumor cells to targeting therapies.

Conversely, under hypoxia or drug pressure, AMPK may promote glycolysis and facilitate resistance. In HCC, sorafenib‐induced metabolic stress activates AMPK, enhances antioxidant capacity, and supports tumor survival.^68^ Sustained AMPK activation under prolonged glycolytic activity inversely correlates with treatment response. This activation appears to occur independently of AMP/ATP levels, suggesting alternative regulatory inputs. AMPK's dual role likely arises from its spatiotemporal regulation of substrate phosphorylation, shifting between tumor‐suppressive and tumor‐promoting effects depending on theTME.^69^ However, the precise mechanisms through which AMPK governs glycolysis and mediates resistance in HCC remain to be fully elucidated.

### 4.2 Cellular myelocytomatosis oncogene

Encoded by the Myc oncogene, c‐Myc is a transcription factor that promotes aerobic glycolysis,^70^ supporting tumor growth even under normoxic conditions. In transgenic mouse livers, c‐Myc overexpression elevates glycolytic enzyme activity and lactate production.^71^ Therefore, future studies are warranted to clarify the regulatory role of c‐Myc in aerobic glycolysis and the potential for treating HCC.

Primarily, c‐Myc regulates glycolytic enzymes GLUT1 and LDHA via multiple mechanisms. For example, the activator of thyroid and retinoid receptor can enhance c‐Myc recruitment to glycolytic gene promoters, promoting sorafenib resistance.^10^ Heat shock protein 90 (HSP90) stabilizes c‐Myc through interaction with ACYP1,^72^ facilitating lenvatinib resistance through the HSP90/MYC/LDHA axis. The nucleolar RNA‐binding protein NOP2, a member of the m5C methyltransferase family, also upregulates c‐Myc and enhances glycolysis through PKM2 and LDHA regulation. NOP2 knockout sensitizes HCC to sorafenib.^73^ Additionally, c‐Myc inhibits HIF‐1α degradation and can synergize with HIF‐1 to activate HK2 and pyruvate dehydrogenase kinase 1, amplifying the Warburg effect.^74^ Nuclear PKM2 further upregulates c‐Myc via β‐catenin,^75^ forming a feedback loop sustaining glycolytic gene expression.

Targeting c‐Myc offers a potential strategy to inhibit hypoxia‐driven glycolysis and tumor growth. Sodium butyrate reduces HK2 via c‐Myc inhibition, enhancing sorafenib efficacy.^76^ Similarly, cleavage and polyadenylation‐specific factor 6 (CPSF6) stabilizes c‐Myc through nuclear colocalization,^77^ while its depletion promotes c‐Myc degradation, inhibits glycolysis, and sensitizes HCC to sorafenib. These findings highlight c‐Myc as a central regulator of metabolism and drug resistance, and support further investigation into its therapeutic targeting in HCC.

### 4.3 Hypoxia‐inducible factor 1

HIF‐1 is a heterodimeric transcription factor composed of an oxygen‐sensitive α‐subunit and a constitutive β‐subunit.^78^ Under normoxic conditions, HIF‐1α is degraded via prolyl hydroxylases (PHDs), but hypoxia inhibits PHD activity, stabilizing HIF‐1α and enabling its nuclear translocation, dimerization with HIF‐1β, and activation of hypoxia‐responsive elements. This activation upregulates GLUT and glycolytic enzymes, enhancing glycolytic flux.^79^ HIF‐1 also induces MCT4 expression to support lactate efflux and pH homeostasis. Chronic hypoxia from early carcinogenesis sustains HIF‐1 activation, with elevated HIF‐1α expression frequently observed in HCC and correlating with poor prognosis.^80^

Beyond hypoxia, HIF‐1α is regulated by AMPK and PI3K/Akt/mTOR pathways.^81^ HSP90 promotes HIF‐1α dimerization,^82^ while pyruvate stabilizes HIF‐1α by inhibiting oxygen‐induced degradation, thereby enhancing glycolytic enzyme expression.^83^ Interestingly, LDHA directly stabilizes HIF‐1α, enhancing its co‐activation of HK2 and PFK1,^84^ revealing an auxiliary role for LDHA in regulating HIF‐1α during aerobic glycolysis.

HIF‐1‐mediated glycolysis is closely linked to sorafenib resistance in HCC. Resistant Huh‐7R cells display increased glucose uptake and lactate production.^85^ Targeting HIF‐1 and its downstream effectors offers therapeutic potential. For example, Simvastatin resensitizes resistant HCC cells to sorafenib via the HIF‐1α/PPARγ/PKM2 axis,^86^ suggesting novel combination strategies. In summary, HIF‐1 not only supports tumor adaptation to hypoxia but also plays a pivotal role in therapeutic resistance.

### 4.4 The PI3K/Akt pathway

The PI3K/Akt pathway is essential for metabolic reprogramming, promoting tumor growth, survival, and drug resistance.^87^ PI3K directly activates Akt, which enhances glycolysis by upregulating enzymes such as HK2, PFK1, and PFKFB.^88^ Akt mediates HK2 interaction with VDAC on the outer mitochondrial membrane, enabling direct ATP utilization to support phosphorylation.^89^ It also promotes the translocation of glucose transporters 1 (GLUT1) and 4 to the membrane, increasing glucose absorption to meet the high metabolic demands of tumor cells.

Furthermore, Akt enhances cell survival by inhibiting p53 and activates mTOR, a serine/threonine kinase that responds to nutrient and growth signals.^90^ mTOR functions within two complexes, mTORC1 and mTORC2. Among these, mTORC1, the main effector of PI3K/Akt signaling, activates HIF‐1α, inducing expression of glycolytic enzymes like LDH and transporters such as GLUT1. Moreover, mTORC1 also enhances GLUT1 translocation and HK2 activity via inhibition of eukaryotic translation initiation factor 4E.^91^ Recent findings highlight the Akt‐dependent regulation of PKM2,^92^ which acts as an upstream modulator of the PI3K/Akt pathway, but yet can activate mTORC1 through interaction with Akt1 substrate 1. In contrast, mTORC2 primarily phosphorylates Akt at Ser473, further upregulating GLUT1, HK2, and PFK1, thereby increasing the glycolysis rate.^93^

Overactivation of the PI3K/Akt pathway is a crucial factor contributing to sorafenib and multidrug resistance in HCC. Inhibiting this pathway can restore drug sensitivity. For instance, LY294002 reverses sorafenib resistance by blocking PI3K/Akt signaling.^94^ Natural compounds such as Rg3, a ginseng‐derived bioactive, downregulate PI3K/Akt signaling and HK2 expression, reducing glycolysis and enhancing sorafenib efficacy in combination therapy.^95^ These findings support the development of PI3K/Akt‐targeted combination strategies to overcome HCC resistance.

### 4.5 p53

The tumor suppressor gene TP53 encodes p53, a key regulator of cellular homeostasis. p53 inhibits c‐Myc under normoxia and HIF‐1α under hypoxia, thereby repressing glycolytic genes, like GLUT1, HK2, and PFKFB3.^96^ Additionally, p53 suppresses MCT1 expression, limiting pyruvate export and glycolytic flux. Moreover, p53 induces TP53‐induced glycolysis regulatory phosphatase (TIGAR),^97^ which degrades F‐2,6‐BP, thereby reducing glycolytic activity and shifting metabolism toward oxidative phosphorylation.

The p53 pathway is frequently dysregulated in tumors. Beyond common TP53 mutations, alternative mechanisms impair p53 function. For instance, mouse double minute 2 homolog inhibits p53 by blocking nuclear translocation, impairing DNA binding, and promoting proteasomal degradation.^98^ Interestingly, fasting can sensitize HCC to sorafenib in a p53‐dependent manner, which is prevented by non‐functional p53 via reduced glucose uptake and impaired pro‐apoptotic signaling.^99^ Restoring wild‐type p53 function remains a therapeutic goal. Oroxylin A enhances p53 phosphorylation and TIGAR expression, suppressing glycolysis in HCC.^100^ Tripartite motif‐containing 37 (TRIM37) enhances sorafenib resistance by regulating p53 ubiquitination and activating glycolysis, identifying TRIM37 as another potential target.^101^ Considering the complexity of the p53 pathway, reactivation strategies must carefully balance its tumor‐suppressive effects with potential toxicity to normal tissues.

## 5 DISCUSSION

Despite advances in targeted therapy for HCC, major drugs like sorafenib and lenvatinib face substantial resistance,^4^ highlighting the urgent need for new therapeutic strategies. Aerobic glycolysis has emerged as a key driver of HCC progression and is closely linked to resistance mechanisms.^60^ This review explores the role of key glycolytic enzymes such as HK2, PFK1, and PKM2 in the metabolism and resistance mechanisms of HCC. These enzymes support tumor growth and can also influence drug metabolism pathways. Additionally, lactate and lactylation modifications contribute to resistance by altering the TME and signal transduction.^58^, ^59^ Crucially, upstream regulators of glycolysis, including AMPK, c‐Myc, and PI3K/Akt, further modulate these processes, collectively contributing to therapeutic resistance. Therefore, a deeper understanding of these regulatory mechanisms offers valuable insight for the development of novel targeted interventions.

The role of AMPK in HCC remains complex and context‐dependent. AMPK exerts both tumor‐suppressive and tumor‐promoting effects depending on cellular context.^102^ While AMPK activation can improve cellular energy balance and suppress tumor proliferation, in certain tumor microenvironments it may facilitate adaptation to stress and promote resistance. Notably, recent findings indicate that AMPK activity status governs the dual functionality of HNF4A in HCC, shifting it from a tumor suppressor under low AMPK activity to a tumor promoter under high activity, thereby linking metabolic sensing to phenotypic plasticity.^103^ This dichotomy reflects the broader paradox of AMPK signaling in cancer—acting as a metabolic checkpoint under physiologic stress while potentially sustaining malignancy under therapeutic pressure. Understanding this dual role is crucial for developing new therapeutic strategies. Particularly in the context of HCC, identifying and targeting agents that can modulate AMPK function to shift it from a tumor‐promoting to a tumor‐inhibiting role presents an exciting and promising research avenue. By thoroughly investigating the mechanisms of AMPK in various tumor environments and its impact on glycolytic pathways, we can better understand its role in resistance and provide novel solutions to overcome targeted drug resistance in HCC.

Lactate and lactylation modifications also warrant further investigation in HCC resistance. First, lactate accumulation not only acidifies the tumor microenvironment to promote tumor growth but also fuels protein lactylation.^52^ Targeting MCTs to reduce lactate accumulation may enhance drug sensitivity. Second, systematic screening and functional validation of lactylation sites, along with characterization of lactylation “writers” and “erasers,” are needed to clarify how lactylation mediates drug resistance through regulation of protein functions and signaling pathways. Lastly, the development of specific inhibitors or activators targeting lactylation modifications should be explored to potentially overcome drug resistance in HCC. Precisely modulating lactylation modifications can enhance the efficacy of targeted therapies and overcome drug resistance.

Emerging technologies are accelerating research into HCC metabolism and resistance. High‐throughput omics and bioinformatics have identified numerous metabolic genes and networks implicated in resistance, offering crucial data support and new directions for the development of metabolic‐targeted therapies. Experimental platforms such as organoids and organ‐on‐a‐chip systems provide physiologically relevant models that better replicate the hepatic tumor microenvironment and disease conditions, enabling more accurate investigation of resistance mechanisms. Future research should focus on identifying glycolysis‐related biomarkers for resistance prediction and treatment monitoring. Additionally, targeting specific isoforms of glycolytic enzymes may improve drug specificity and efficacy. Integrating these advanced technologies and research strategies will contribute to a more comprehensive understanding of metabolic regulation in HCC, providing more precise and effective solutions for personalized treatment.

To strengthen clinical feasibility, future research must prioritize personalized metabolic interventions and rational combination strategies. Personalized approaches could involve: (1) profiling a patient's metabolic landscape at diagnosis (via omics and imaging) to identify dominant glycolytic drivers; (2) selecting therapies targeting these drivers (e.g., PKM2 inhibitors for PKM2‐high tumors, AMPK modulators for context‐specific AMPK activity); and (3) monitoring response via dynamic biomarker tracking to adjust therapies as resistance evolves. Combination strategies could pair metabolic inhibitors with existing agents: for example, glycolysis inhibitors to sensitize tumors to lenvatinib by reducing ATP production, or lactylation modulators to enhance immunotherapy efficacy by normalizing TME acidity and reducing immunosuppression. Additionally, combining metabolic targets with targeted therapies or immunotherapies could address parallel resistance pathways, preventing escape.

In summary, integrating mechanistic insights into glycolysis, AMPK, and lactylation with advanced technologies and personalized strategies bridges the gap between basic research and clinical practice. By prioritizing context‐aware, biomarker‐driven interventions and rigorously testing combination therapies in translational models, we can move toward more effective, personalized solutions to overcome HCC drug resistance. This approach not only reinforces the clinical relevance of metabolic targeting but also provides a clear roadmap for future research and therapeutic development.

## AUTHOR CONTRIBUTIONS

**Longtao Zhao:** Conceptualization; writing – review and editing; writing – original draft; methodology; visualization. **Junjie Cheng:** Investigation; validation. **Yiming Zheng:** Software; methodology. **Jing Wu:** Investigation. **Jia Fan:** Validation; supervision. **Haixiang Sun:** Supervision; validation; methodology. **Chao Gao:** Methodology; validation; supervision.

## CONFLICT OF INTEREST STATEMENT

The authors declare no conflicts of interest.
